# *Arabidopsis *plants grown in the field and climate chambers significantly differ in leaf morphology and photosystem components

**DOI:** 10.1186/1471-2229-12-6

**Published:** 2012-01-11

**Authors:** Yogesh Mishra, Hanna Johansson Jänkänpää, Anett Z Kiss, Christiane Funk, Wolfgang P Schröder, Stefan Jansson

**Affiliations:** 1 Umeå Plant Science Centre, Department of Plant Physiology, Umeå University, SE-901 87 Umeå, Sweden; 2 Umeå Plant Science Centre, Department of Chemistry, Umeå University, Umeå, Sweden

**Keywords:** *Arabidopsis thaliana*, Carotenoids, Chlorophyll fluorescence, Early light inducible proteins (ELIPs), Field Plants, Indoor Plants, Light harvesting proteins (LHCs)

## Abstract

**Background:**

Plants exhibit phenotypic plasticity and respond to differences in environmental conditions by acclimation. We have systematically compared leaves of *Arabidopsis thaliana *plants grown in the field and under controlled low, normal and high light conditions in the laboratory to determine their most prominent phenotypic differences.

**Results:**

Compared to plants grown under field conditions, the "indoor plants" had larger leaves, modified leaf shapes and longer petioles. Their pigment composition also significantly differed; indoor plants had reduced levels of xanthophyll pigments. In addition, Lhcb1 and Lhcb2 levels were up to three times higher in the indoor plants, but differences in the PSI antenna were much smaller, with only the low-abundance Lhca5 protein showing altered levels. Both isoforms of early-light-induced protein (ELIP) were absent in the indoor plants, and they had less non-photochemical quenching (NPQ). The field-grown plants had a high capacity to perform state transitions. Plants lacking ELIPs did not have reduced growth or seed set rates, but their mortality rates were sometimes higher. NPQ levels between natural accessions grown under different conditions were not correlated.

**Conclusion:**

Our results indicate that comparative analysis of field-grown plants with those grown under artificial conditions is important for a full understanding of plant plasticity and adaptation.

## Background

Much of our understanding of plant growth, development and metabolism has come from studies--often using *Arabidopsis thaliana *as a model system--based on laboratory-grown specimens. Nevertheless, plants exhibit huge phenotypic plasticity and respond to differences in environmental conditions by acclimation [see for example [1,2]], hence environmental conditions greatly influence the outcome of studies. Field studies are generally rare because (*inter alia*) the photoperiod, temperature and light intensity are not controlled and growth conditions are difficult to reproduce. However, in a few studies *Arabidopsis *grown in natural environments has been used to study, for example, reproductive timing, fitness-related quantitative traits and flowering time [e g [3-6]]. The main rationale for performing experiments under controlled conditions in growth cabinets or climate chambers is to minimize variations in measured traits apart from those due to applied treatments. However, even in the laboratory conditions are likely to vary to some extent, thus experimental results obtained using different brands of climate chambers, different standard procedures and different equipment in different laboratories are also likely to vary to some degree. The variations in the field are much greater, but few authors acknowledge that acquired results are strongly influenced by the growth conditions employed, and even fewer consider how the results may have differed had the experiments been performed under conditions that plants are actually adapted to, i.e. variable field conditions. Thus, the emphasis on controlling growth parameters to allow comparative investigation of plant physiology can provide valuable information, but it also constrains our understanding of how plants adapt to field conditions.

Due to the limitations outlined above, there is a need for comprehensive investigations of field-grown specimens to evaluate phenotypic characteristics expressed in plants grown under natural conditions, in which conditions are not controlled. We have therefore developed procedures and tools for analyzing field-grown *Arabidopsis *plants, including mutants and transgenics, in "semi-natural" conditions [7]. We have also shown that mutants exhibiting no obvious phenotypic variation under laboratory conditions can suffer significant loss of fitness [8]. For these reasons, studies on field-grown *Arabidopsis *(e.g. using high-throughput DNA microarray and metabolomics techniques) may be more informative for assessing plants' responses in real environments than those performed under controlled conditions [9]. This raises complex problems, since a key characteristic of field conditions is that they vary in unpredictable ways, resulting in phenotypic variations among field-grown plants in each experiment, even at the same site. Responses to different plant ecotypes adapted to different environments are also likely to vary significantly at field sites. Nevertheless, failure to address these problems will inevitably constrain our understanding of plant responses.

Leaf traits, including those relating to photosynthesis, have particularly plastic responses to the growth environment. Various leaf acclimation responses have been recorded at many levels, from whole-plant morphology down to the stoichiometry of the photosynthetic apparatus [10,11], for example, adjustments in reaction center stoichiometry and Rubisco levels [12,13]. Pronounced changes in response to environmental variations have been well documented in levels of photosynthetic antenna, i e pigments and pigment-binding light-harvesting chlorophyll-binding (LHC) antenna proteins, Lhca protein and Lhcb proteins associated with photosystems I (PSI) and II (PSII), respectively, and in other members of the light-harvesting chlorophyll-binding (LHC) "superfamily", notably PsbS [14] and early light induced proteins (ELIPs) [15,16]. Changes in light-harvesting pigments and proteins influence several photosynthetic parameters, e.g. the capacity for qE energy-dependent non-photochemical quenching (NPQ) or feedback de-excitation, which harmlessly dissipates excess absorbed light energy as heat, and the xanthophyll cycle (XC) pool size differs both between species [17,18] and during acclimation [19-21]. Much less is known about how the light regime influences so-called state transitions in which the excitation energy inputs into the two photosystems [22] are balanced by reversible phosphorylation of the LHC proteins catalyzed by Stn7 kinase [23] and Pph1 phosphatase [24]. However, this process too may be profoundly affected by environmental variations in the field.

A comparative analysis of *Arabidopsis *plants grown under various light intensities has been published [13], but the "high light" conditions used in this and other laboratory studies, typically 600-800 μmol quanta m^-2 ^s^-1^, are equivalent to rather "low light" in the field, where light intensities on sunny days can exceed 2000 μmol quanta m^-2 ^s^-1^. A systematic comparison between *Arabidopsis *plants grown in the laboratory with those grown under field conditions could therefore be informative for optimizing field-growth and reproducibility in future experiments. Hence, in the study presented here we examined *Arabidopsis *plants grown under three different light intensities in climate chamber conditions and related the magnitude of differences among them to those observed in field-grown plants. We also examined in more detail than previously changes in a number of regulatory processes whose importance our previous data suggest could be over- or under-estimated when analyzed plants are grown under "unnatural laboratory conditions" in terms of light intensity and lack of fluctuations in light and temperature. We used field-grown plants as references, as we believe that they best reflect the status of plants under the growth conditions to which *Arabidopsis *is adapted. Our comparison shows that *Arabidopsis *plants in climate chambers are similar in many respects to those grown in the field, but we also pinpoint some parameters for which extrapolating results from analyses of plants--in particular those grown under short day (SD) photoperiods--in controlled conditions to plants grown under natural conditions could be misleading.

## Results

### Plants grown indoors have enlarged leaves, different leaf shapes and longer petioles

**Figure 1:**
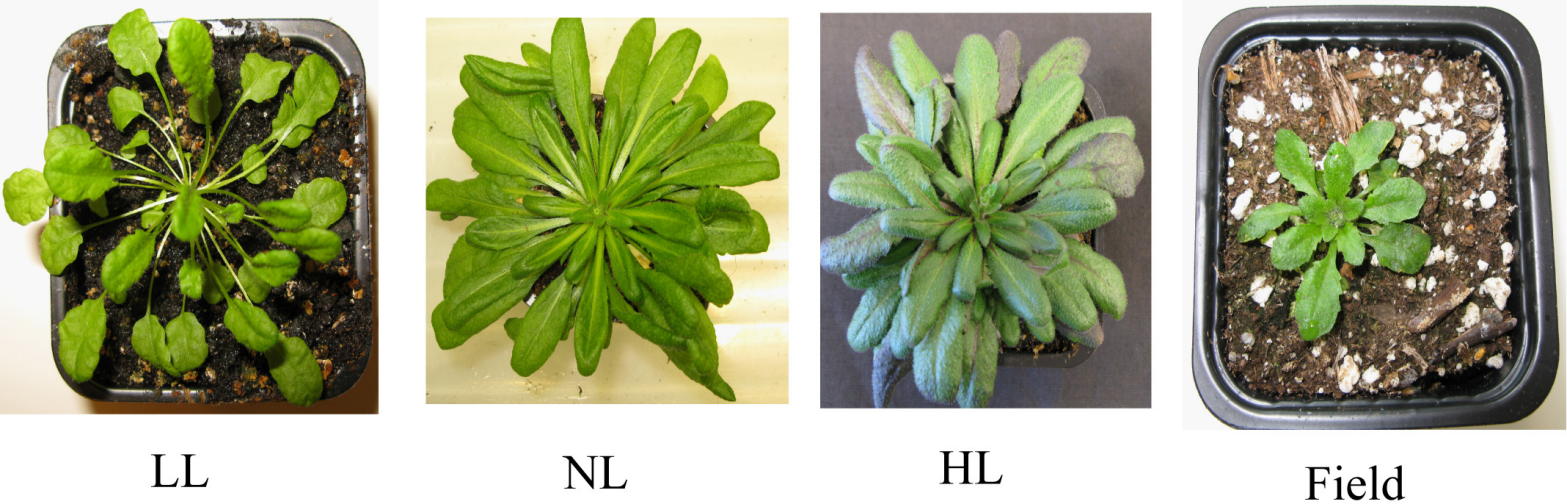
**Phenotypic plasticity of *Arabidopsis thaliana *rosettes in different growth regimes**. Plants were grown in climate chambers under Low, Normal or High Light (LL, NL and HL; 30, 300 and 600 μmol quanta m^-2 ^s^-1^, respectively) and under field conditions.

**Figure 2:**
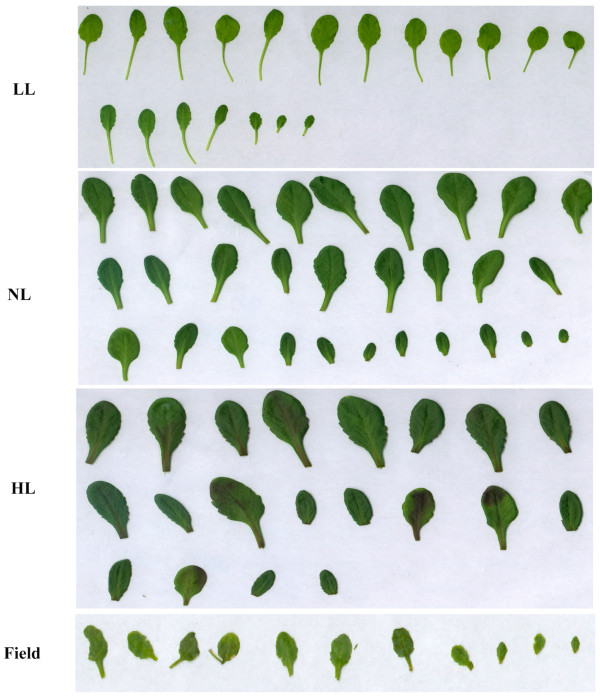
**Rosette leaf complements of *Arabidopsis thaliana *plants under different growth regimes**. Plants were grown in climate chambers under Low, Normal or High Light (LL, NLand HL; 30, 300 and 600 μmol quanta m^-2 ^s^-1^, respectively) and under field conditions.

**Figure 3:**
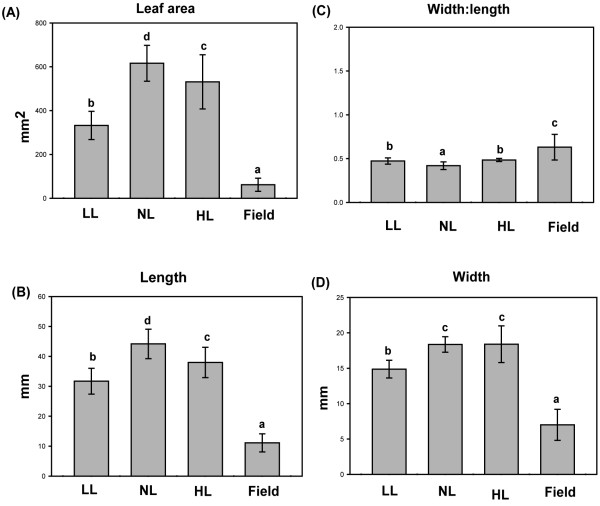
**Variation in leaf traits of *Arabidopsis thaliana *plants under different growth regimes**. Plants were grown in climate chambers under Low, Normal or High Light (LL, NL and HL; 30, 300 and 600 μmol quanta m^-2 ^s^-1^, respectively) and under field conditions. **A **Leaf area (mm^2^), **B **length (mm), **C **width:length ratio, **D **width (n = 10). Different lower case letters above bars indicate significant differences (*p *< 0.001), according to separate Duncan's new multiple range tests followed by contrast analysis (indoor vs. field plants) applied to data presented in each column.

Leaf size and shape are known to respond to changes in light levels [25] and in this study we found that leaves of the *Arabidopsis *plants grown indoors under low (LL), normal (NL) and high (HL) and field-grown plants showed clear differences in morphology (Figure 1). Indoor plants were grown under short days (SD) so bolting time was strongly dependent on their growth rate; plants grown under low light bolted later than those grown at higher irradiance. The LL plants were characterized by longer petioles (Figure 2), less compact rosettes and took 12 weeks to reach maturity, while NL plants took only 5 weeks. Leaves from the plants grown under HL were larger and thicker than those grown under LL and NL. Moreover, HL, and to some extent NL, plants tended to have more curled leaf edges and a more reddish appearance--certainly because of accumulation of anthocyanin [26]--than LL plants. HL plants also had shorter petioles and smaller leaf rosettes. The field-grown plants, pre-grown in climate chambers, took less than 3 weeks in the field to bolt. These plants were characterized by shorter petioles and even smaller rosettes than the plants grown under HL conditions. To quantify these differences, we analyzed leaf physiognomy using digital image processing. The most notable difference was in leaf area between field-grown plants and those grown under all indoor light environments (Figure 3A), leaves of field-grown plants being significantly smaller than those grown under all growth chamber conditions (as revealed by contrast analysis of indoor vs field plants, *p *< 0.001). LL leaves were significantly smaller (*p *< 0.001) than both NL and HL leaves, which also differ significantly from each other (as revealed by *post-hoc *analysis). Field-grown leaves also differed in width:length ratio (contrast analysis, *p *< 0.001) from those grown under all indoor light conditions (Figure 3C). Correspondingly, leaf length (Figure 3B) and width (Figure 3D) showed the same trends observed for leaf area. Both photoperiod and light intensity could influence these differences (see below).

### Indoor plants have much less xanthophyll cycle pigments

**Figure 4:**
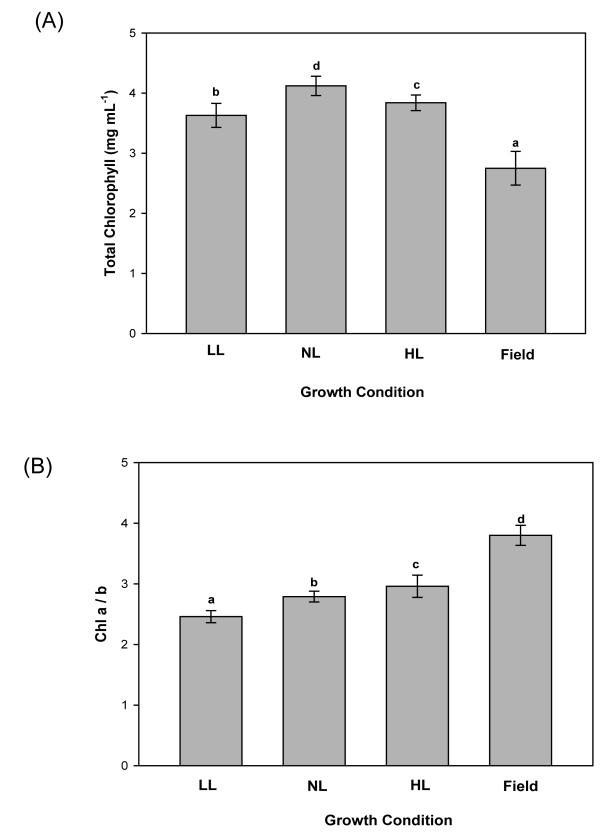
**Variation in chlorophyll in *Arabidopsis thaliana *leaves in different growth regimes**. Plants were grown in climate chambers under Low, Normal or High Light (LL, NL and HL; 30, 300 and 600 μmol quanta m^-2 ^s^-1^, respectively) and under field conditions. **A **Total chlorophyll content and **B **Chl a/b ratio were determined from leaf discs (n = 3 pools of leaves each from 5-15 plants). Different lower case letters above bars indicate significant differences (*p *< 0.001), according to separate Duncan's new multiple range tests followed by contrast analysis (indoor vs. field plants) applied to data presented in each column.

Chlorophyll content per unit area was significantly lower in leaves of field-grown plants than in leaves of all three types of indoor-grown (LL, NL and HL) plants (Figure 4A), but their Chl a/b ratio was much higher (Figure 4B). The carotenoid contents also differed between the plants (Table 1). Lutein levels were lower in indoor plants, and neoxanthin levels somewhat lower. However, the greatest reductions were in their xanthophyll cycle (XC) pool size (the sum of violaxanthin, antheraxanthin and zeaxanthin); not only in LL and NL but also in HL grown plants, which had 30% less V+A+Z pigments than field-grown plants. In addition to differences in pool size, there were also differences in the de-epoxidation state (DES) of the XC pool (Table 1); leaves taken from field-grown plants had higher DES levels than all the indoor grown plants. It appears therefore that both the alpha- and beta-branches of the carotenoid biosynthetic pathway were affected by the growth conditions, and adjustments of metabolic fluxes resulted in the synthesis in the field-grown plants.

**Table 1:** Carotenoid contents of *Arabidopsis *grown indoors under low light (LL), normal light (NL) and high light (HL) conditions, and in the field

Pigment	LL	NL	HL	Field
Neoxanthin	1.4 ± 0.3^b^	1.0 ± 0.2^a^	1.1 ± 0.1^a^	1.5 ± 0.1^b^

Violaxanthin	1.4 ± 0.2^a^	1.6 ± 0.1^b^	2.5 ± 0.1^c^	8.5 ± 0.2^d^

Anthraxanthin	0 ± 0	0 ± 0	0.24 ± 0.1^a^	1.4 ± 0.1^b^

Zeaxanthin	0 ± 0	0 ± 0	0 ± 0	1.3 ± 0.1^a^

VAZ	1.4 ± 0.1^a^	1.6 ± 0.1^b^	2.74 ± 0.1^c^	11.2 ± 0.1^d^

DES	0	0	0.043^a^	0.18^b^

Lutein	13.5 ± 0.9^b^	12.4 ± 0.4^a^	15.3 ± 0.1^c^	28.1 ± 0.2^d^

beta-carotene	2.3 ± 0.2^c^	2.4 ± 0.1^b^	1.4 ± 0.1^a^	1.4 ± 0.1^a^

Values are percentages of total chlorophyll. Data presented as means ± SD (n = 3). Different lower case letters indicate significant differences (*p *< 0.001), according to separate Duncan's new multiple range tests followed by contrast analysis (indoor *vs*. field plants) applied to data presented in each column.

### Lhca5, a component of PSI antenna complex, is significantly reduced in field-grown plants

**Figure 5:**
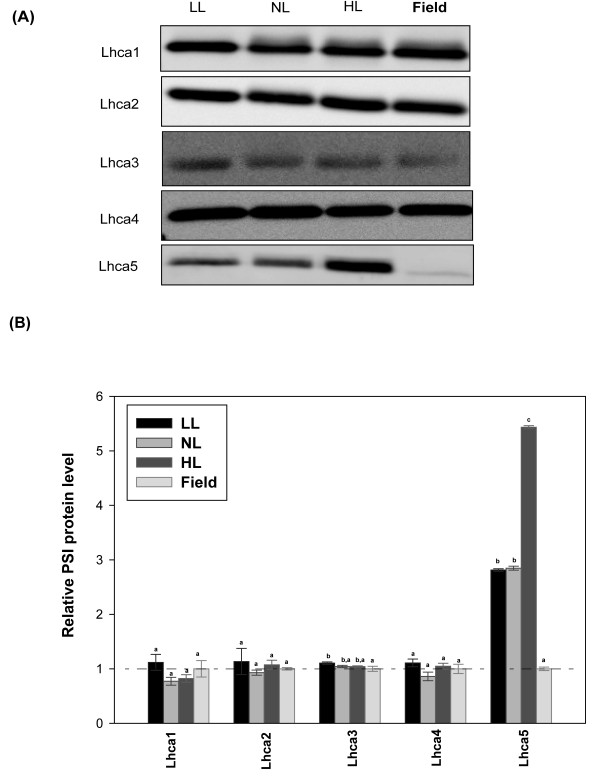
**PSI protein composition of *Arabidopsis thaliana *in different growth regimes**. Plants were grown in climate chambers under Low, Normal or High Light (LL, NL and HL; 30, 300 and 600 μmol quanta m^-2 ^s^-1^, respectively) and under field conditions. **A **Results of immunoblot analysis of thylakoid membranes, probed with antibodies against Lhca1, Lhca2, Lhca3 Lhca4 and Lhca5. Lanes were loaded with 1.0 μg chlorophyll. **B **Quantification of immunoblot data. *Error bars *indicate SE (n = 3 pools of leaves each from 5-15 plants), the relative abundances of proteins were normalized to the data for field-grown plants. Different lower case letters above bars indicate significant differences (*p *< 0.001), according to separate Duncan's new multiple range tests followed by contrast analysis (indoor vs. field plants) applied to data presented in each column.

We also compared levels of pigment-binding proteins in the indoor- and field-grown plants since, theoretically, the significant differences in pigment composition between the plants should have been reflected in levels of these proteins. Figure 5A shows results of an immunoblot analysis of levels of five light harvesting antenna proteins (Lhca1-5) of photosystem I (PSI) in indoor (LL, NL and HL) and field-grown plants. To highlight the differences in levels of each protein we normalized data for relative band intensities in indoor plants against our data for field-grown plants (Figure 5B). The levels of Lhca1, Lhca2, Lhac3 and Lhca4 showed little or no changes relative to those of field-grown plants (Figure 5B). However, Lhca5--a protein present in substoichiometric amounts [27]--accumulated in plants grown under all indoor conditions, especially in HL plants.

### Indoor plants accumulate high levels of Lhcb1 and Lhcb2

**Figure 6:**
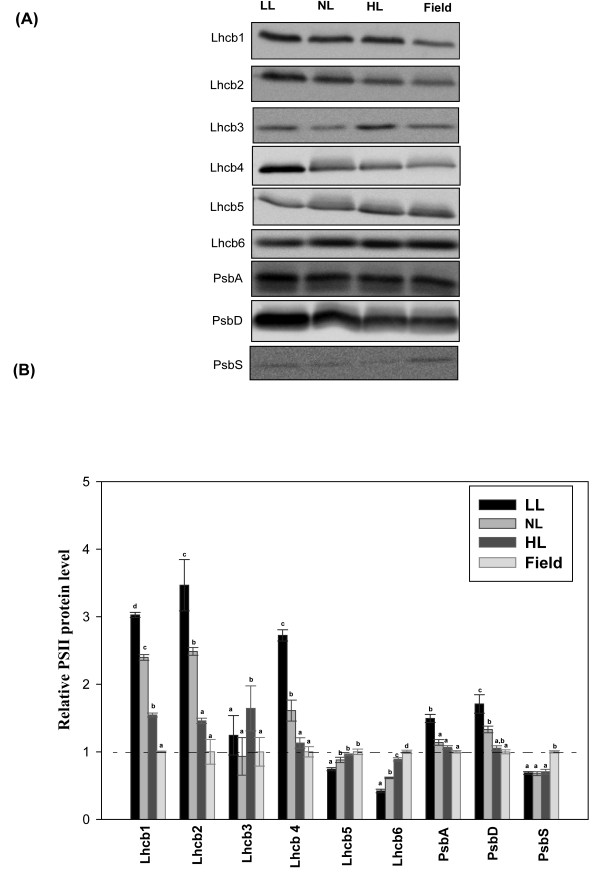
**PSII protein composition of *Arabidopsis thaliana *in different growth regimes**. Plants were grown in climate chambers under Low, Normal or High Light (LL, NL and HL; 30, 300 and 600 μmol quanta m^-2 ^s^-1^, respectively) and under field conditions. **A **Results of immunoblot analysis of thylakoid membranes probed with antibodies against Lhcb1, Lhcb2, Lhcb3, Lhcb4, Lhcb5, Lhcb6, PsbA (D1), PsbD (D2) and PsbS. Lanes were loaded with 1.0 μg chlorophyll. **B **Quantification of immunoblot data. Error bars indicate SE, n = 3 pools of leaves each from 5-15 plants. The relative abundances of peptides were normalized to the data for field-grown plants. Different lower case letters above bars indicate significant differences (*p *< 0.001), according to separate Duncan's new multiple range tests followed by contrast analysis (indoor vs. field plants) applied to data presented in each column.

The relative levels of Lhcb1, Lhcb2 and Lhcb3, constituting the major light harvesting chlorophyll a/b-binding proteins of the PSII antenna (LHCII), are known to vary with growth conditions; lower amounts of these proteins being present in leaves of HL-treated plants than those of LL-treated plants [28]. When the levels of Lhcb1, Lhcb2 and Lhcb3 were measured in indoor- and field-grown *Arabidopsis*, a strong pattern was observed; LL plants had more than 3-fold, NL plants more than 2-fold and HL plants ca. 50% higher levels of Lhcb1 and Lhcb2 compared with field-grown plants, while the level of Lhcb3 was unchanged (Figure 6A). Lhcb4, Lhcb5 and Lhcb6 (the minor light-harvesting components of LHCII), also showed distinct patterns of accumulation in indoor- and field-grown plants. The Lhcb4 level was higher in indoor plants (LL, NL and HL) with maximum accumulation in LL-plants (Figure 6B). In contrast, Lhcb5 and Lhcb6 levels were decreased in indoor plants. An inherent problem in comparisons of photosynthetic proteins between plants grown under different conditions is in quantitatively relating levels of these proteins between them. Relating protein levels to chlorophyll content is the most robust method for displaying such comparative data, but in the types of conditions we investigated, which induce variations in pigment levels, data interpretation is less straightforward. We normalized data for relative band intensities in indoor plants against our data for field-grown plants. Both PSII reaction centre proteins D1 and D2 were, on chlorophyll basis, slightly more abundant in indoor plants (Figure 6A), but the changes in levels of the LHC proteins mentioned above were more pronounced in all cases, indicating that the level of LHC proteins per PSII differed amongst the plants. We also measured levels of PsbS, a protein that regulates the photo-protective thermal dissipation process of qE. The level of PsbS was found to be significantly reduced in all indoor conditions (contrast analysis indoor vs field plants, *p *< 0.001), compared to field plants (Figure 6B).

### Early light-induced proteins (ELIPs) are absent from indoor-grown plants

**Figure 7:**
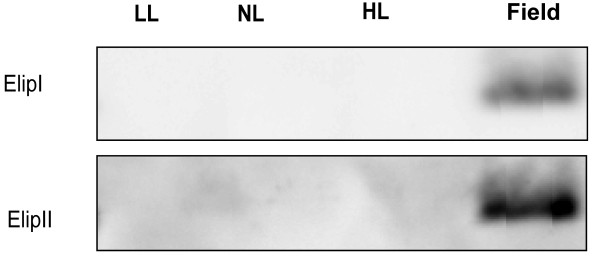
**ELIPI and ELIPII content of *Arabidopsis thaliana *in different growth regimes**. Plants were grown in climate chambers under Low, Normal or High Light (LL, NL and HL; 30, 300 and 600 μmol quanta m^-2 ^s^-1^, respectively) and under field conditions. Results of immunoblot analysis of thylakoid membranes probed with antibodies against ELIPI and ELIPII. Lanes were loaded with 1.0 μg chlorophyll. n = 3 pools of leaves each from 5-15 plants.

Early light induced proteins (ELIPs), belonging to the LHC super-gene family, accumulate transiently in plants exposed to high light intensities [29] and are postulated to protect plants from photo-oxidative stress [30]. ELIP protein accumulation has also been used as an indicator of light stress; the proteins reportedly accumulate to much higher levels in pea plants grown in the field than in counterparts grown indoors [31]. We corroborated these findings and found that both ELIP gene products present in *Arabidopsis*, ELIP I and ELIP II, were only detectable in field-grown plants (Figure 7). The amount of ELIP in field-grown plants was much more variable, compared to other proteins, between batches of plants grown at different occasions, but ELIPs were apparently absent in plants grown under all the applied indoor conditions.

### Indoor plants have a much-reduced NPQ (non-photochemical quenching) capacity

**Figure 8:**
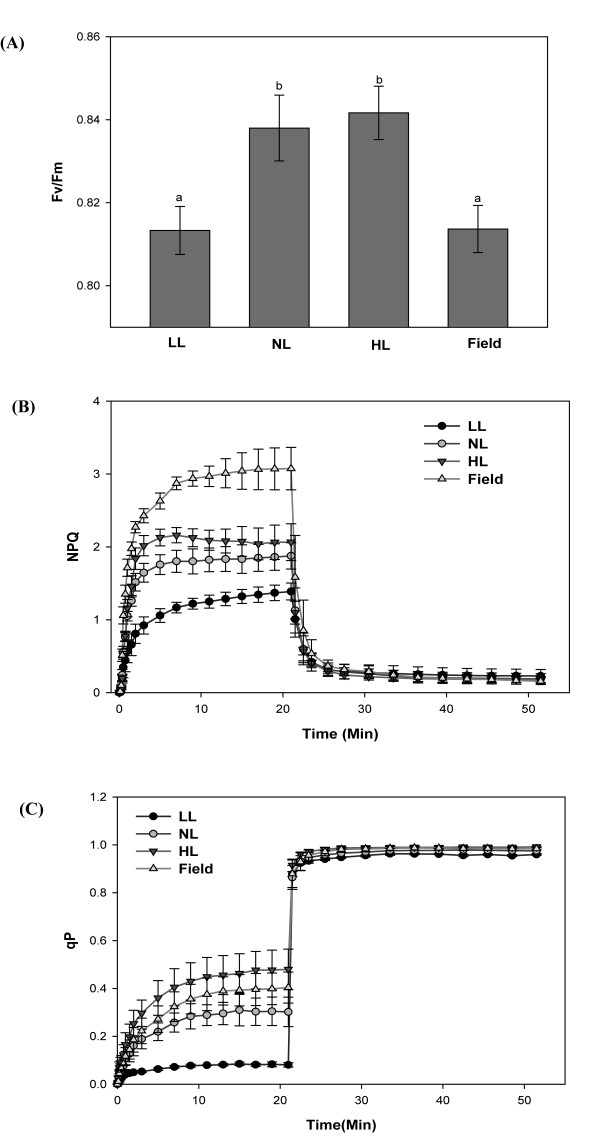
**Photosynthetic functions in *Arabidopsis thaliana *in different growth regimes**. Plants were grown in climate chambers under Low, Normal or High Light (LL, NL and HL; 30, 300 and 600 μmol quanta m^-2 ^s^-1^, respectively) and under field conditions. Photosynthetic function was assessed in dark-adapted leaves. Light response curves are shown for (**A**) Fv/Fm (**B**) non-photochemical quenching (NPQ) and (**C**) qP. Data represent means ± SE for leaves from at least six plants grown in two batches. Different lower case letters above bars indicate significant differences (*p *< 0.001), according to separate Duncan's new multiple range tests followed by contrast analysis (indoor vs. field plants) applied to data presented in each column.

In order to analyze photosynthetic functions of indoor plants more comprehensively we compared LL-, NL-, HL- and field-grown plants using chlorophyll fluorescence analysis, which has been widely used for monitoring photosynthetic functions in plants grown under both indoor and field conditions [32]. The results are shown in Figure 8. Detailed descriptions of the photoinhibition properties of plants with different prehistories have been previously published. The fluorescence parameter Fv/Fm (see [32] for definition) is a particularly dynamic variable, which can change rapidly when plants are shifted to different environments, and light conditions are known to influence Fv/Fm values particularly strongly [32]. When measured under similar conditions (after dark adaption), our plants showed small differences in their Fv/Fm levels, with HL plants exhibiting the highest ratios (Figure 8A). Under constant indoor conditions little or no photoinhibition was induced, but HL plants had a greater capacity to cope with high light intensities. It is possible that our field-grown plants had an even higher capacity, but since they were taken from the field before dark adaptation, photoinhibition of photosynthesis had already developed and their Fv/Fm ratios were therefore slightly lower than those of indoor NL and HL plants. The much reduced capacity of indoor - in particular LL - plants to perform NPQ (Figure 8B) was probably a consequence of their low levels of XC pigments and PsbS. The qP value was highest for HL plants; q*P *values for field-grown plants were not significantly different from those of LL and NL plants (Figure 8C).

### Field-grown plants containing small amounts of Lhcb1 and Lhcb2 still perform state transitions

Since the plants grown in the field had much lower contents of Lhcb1 and Lhcb2 than plants typically used for studies of state transitions (i.e. indoor plants), an intriguing question is whether field grown plants have sufficient LHCII for efficient state transitions, especially since the fraction of LHCII that can be phosphorylated during state transitions is often regarded as "peripheral". Therefore, we quantified the capacity to perform state transitions in the field-grown plants and those grown under the three light treatments indoors (Table 2), fluorescence traces are shown in Additional file 1: Figure S1. As indicated in the methods section, these measurements were performed on separate batches of plants to those used for most of the other analyses. However, their chlorophyll levels and chl a/b ratios were similar (2.75 ± 0.28 and 3.8 ± 0.17 vs. 2.68 ± 0.23 and 3.77 ± 0.11, respectively) to those of plants analyzed in greater detail, suggesting that their antenna sizes were similar since chl a/b ratio is an indicator of antenna size [33]. Interestingly, field-grown plants had a very high capacity for performing state transitions, with the calculated qT parameter being highest for field-grown plants. However, if state transitions were calculated using Fs instead of Fm ("qS"), no significant differences were detected (Table 2). The results of the fluorescence analyses (Additional file 1: Figure S1) suggested that the specific measuring conditions used might have induced some NPQ. The Fm' value after the first round of light activation was much lower than Fm, and qT values were rather low. Nevertheless, the fraction of LHCII found in field-grown plants was clearly sufficient for state transitions.

**Table 2:** State transition parameters in LL, NL, HL and field grown plants

Parameters	LL	NL	HL	Field
**qS**	0.69 ± 0.015^a^	0.79 ± 0.016^c^	0.73 ± 0.05^b^	0.72 ± 0.08^b^

**qT**	0.017 ± 0.005^a^	0.045 ± 0.001^a, b^	0.049 ± 0.014^b^	0.058 ± 0.011^b^

**t_1/2_(s)**	146.61 ± 7.41^a^	197.69 ± 0.85^b^	158.46 ± 1.30^c^	115.0 ± 4.35^d^

Data presented as means ± SD (n = 3). Different lower case letters indicate significant differences (*p *< 0.001), according to separate Duncan's new multiple range tests followed by contrast analysis (indoor *vs*. field plants) applied to data presented in each column

### Photoperiod is the main determinant of leaf size and shape, other factors are more important for photosynthetic traits

**Figure 9:**
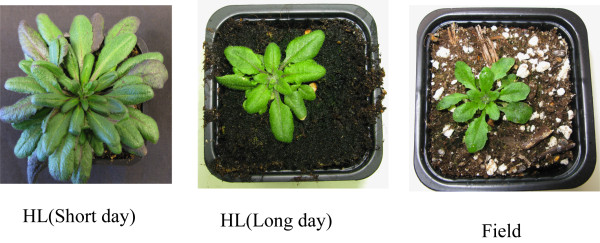
**Phenotypic plasticity of *Arabidopsis thaliana *in different growth regimes**. Plants were grown in climate chambers under high light (600 μmol quanta m^-2 ^s^-1^) with 9 h (SD) or 16 hour (LD) photoperiods and under field conditions.

An important issue is whether the observed differences were simply caused by the difference in photoperiod SD vs. LD in indoor and field conditions, respectively, or if other environmental factors (e g variations in light, wind, and biotic interactions) were more important. To address this issue, we selected the parameters we had found to mainly differentiate plants grown indoors and in the field. These were used to compare plants grown in growth chambers under HL in SD and LD (16 h photoperiods) and field-grown plants. In terms of many measured parameters, the LD plants were intermediate between the SD- and field-grown plants, but an obvious pattern was observed. The overall growth phenotype--including flowering time (not shown)--was largely determined by photoperiod (Figure 9 and Table 3), since in terms of leaf area, length, width and leaf width:length ratio, LD plants were all more similar to the field-grown plants than to SD-plants. The chl a/b ratio of LD plants was also closer to that of the field-grown plants than to SD plants. However, the photosynthetic parameters--amounts of Lhca5, ELIP I and II; Fv/Fm; NPQ; qE; qS; qT and t(1/2)--of LD plants were all more similar to those of the SD plants than to the field-grown plants (Table 3). Clearly, factors other than photoperiod were the strongest determinants of the variations in photosynthetic traits between indoor and field-grown plants.

**Table 3:** Variation in chlorophyll, leaf traits, Lhca 5, ELIP I, ELIP II, non-photochemical quenching (NPQ) and state transition parameters

Parameters	HL (short day)	HL(long day)	Field
Leaf area (mm^2^)	531.28 ± 123.73	175.608 ± 40.40	61.75 ± 30.01

Leaf length (mm)	37.96 ± 5.05	20.76 ± 2.65	11.10 ± 3.04

Leaf width (mm)	18.39 ± 2.59	11.55 ± 1.24	7.0 ± 2.1

Leaf width: Leaf length ratio	0.48 ± 0.017	0.56 ± 0.03	0.63 ± 0.15

Chlorophyll content μg cm^-2^	28.23 ± 2.96	15.23 ± 0.57	18.76 ± 0.23

Chl a/b	2.96 ± 0.18	3.56 ± 0.18	3.70 ± 0.10

Lhca5	38.49 ± 2.20	33.93 ± 1.30	9.36 ± 0.65

ELIP I	0.0 ± 0.0	0.0 ± 0.0	63.14 ± 3.34

ELIPII	0.0 ± 0.0	0.0 ± 0.0	22.77 ± 2.45

Fv/Fm	0.84 ± 0.005	0.84 ± 0.007	0.81 ± 0.009

NPQ	2.15 ± 0.13	2.53 ± 0.29	3.10 ± 0.25

qE	1.97 ± 0.11	2.35 ± 0.12	2.92 ± 0.23

qS	0.73 ± 0.05	0.69 ± 0.02	0.72 ± 0.08

qT	0.049 ± 0.014	0.044 ± 0.004	0.058 ± 0.011

t_1/2_(s)	158.46 ± 1.30	166.05 ± 1.91	115.0 ± 4.35

Plants grown in climate chambers under high light (600 μmol quanta m^-2 ^s^-1^) short day (SD), long day (LD) and under field conditions. Data presented as means ± SD (n ≥ 3)

### ELIPs are dispensable under field conditions

*Arabidopsis *mutants affected in xanthophyll synthesis and metabolism have been extensively studied by our research group [8] and others [34], but mutants lacking ELIPs have been less thoroughly characterized. However, in a recent study a double knock out (KO) mutant lacking both ELIP proteins, ELIP1 and ELIP2, was generated. This mutant did not exhibit obvious phenotypic deviations from wild-type in growth traits when grown under photoinhibitory conditions [35]. In the light of findings by us and others [31] that ELIPs can accumulate to high levels in field-grown plants, we analyzed growth and silique production of the ELIP double mutant in the field in two different years. In terms of both growth and visible phenotype, double ELIP mutants were indistinguishable from wild-type plants. In 2008 the number of siliques produced by wild type-plants and the ELIP double mutant were not significantly different (201 ± 19 and 178 ± 14, respectively), but the ELIP double mutants had a lower survival rate in the experiment (57%) compared to wild-type plants (83%). In 2009, neither survival (100 vs. 93%) nor the number of siliques (11.6 ± 1.4 vs. 14.3 ± 1.7) differed significantly between wild-type and ELIP double mutants.

### NPQ levels in *Arabidopsis *accessions grown in the field and indoors are not correlated

**Figure 10:**
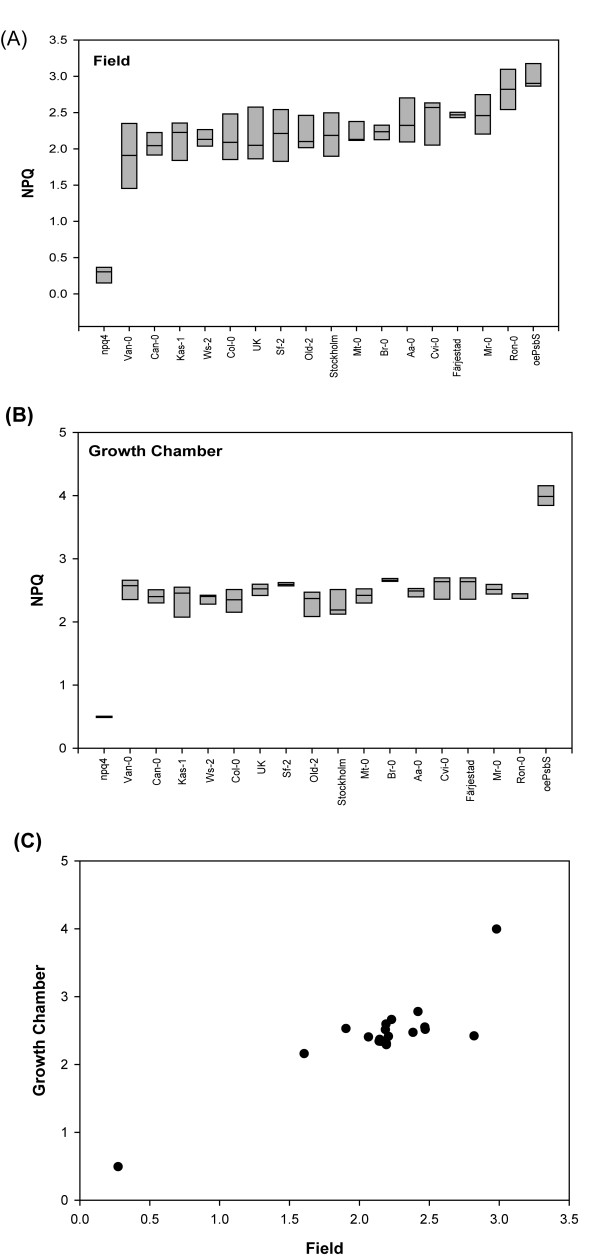
**Variation in non-photochemical quenching (NPQ) in natural *Arabidopsis *accessions, a PsbS mutant (*npq4*) and a PsbS overexpresser (*oePsbS*)**. **A **In the field and **B **in a growth chamber. **C **Correlation (scatter plot) between NPQ levels for the accessions grown in growth chambers and in the field. Data represent means ± SE for leaves from at least six plants grown in two batches.

Further issues that warrant attention are the extents to which plasticity in photosynthesis traits varies between different *Arabidopsis *accessions and may influence the results of genetic studies. To address these issues, we selected *Arabidopsis *accessions that have been previously found to have particularly high or low levels of NPQ [36]. In addition, we included two Swedish accessions, as well as the well-characterized *npq4 *mutant, which lack PsbS and hence have very low levels of NPQ [37], and a transgenic overexpressing PsbS (*oePsbS*) that shows approximately two-fold enhancement of NPQ under lab conditions [38]. When NPQ levels of this set of genotypes grown in the field (Figure 10A) and indoors (Figure 10B) were compared, the results were strikingly different. As expected, the within-accession variation was higher in the field than in the lab. However, the between-accession variation was also much larger in the field and one natural accession, Ron-0, showed almost as much NPQ under field conditions as *oePsbS*, although (intriguingly) Ron-0 was one of the accessions selected for having a particularly low NPQ level. When the NPQ values we measured for the natural accessions in the field were plotted against values measured for plants grown indoors, no correlation was found except that those of *npq4 *and *oePsbS *were extremes (Figure 10C).

## Discussion and Conclusions

In recent decades our understanding of the molecular basis of photosynthesis has increased impressively. It is increasingly evident that the fundamental structure of the photosynthetic apparatus is an example of the capacity for complex, highly sophisticated systems to evolve, since cyanobacteria, green algae and higher plants (which apparently diverged hundreds of millions of years ago) have very similar photosynthetic machineries [39,40]. The main differences in the photosynthetic apparatus of these taxa are in the "peripheral" parts, such as the antenna systems. For example, the phycobilisomes of cyanobacteria have been replaced with the LHC proteins in green algae and higher plants, and there are wide variations in their photosynthetic pigments, many of which were previously used as key taxonomic descriptors. Much regulation is exerted by the antenna systems, where the qE type of NPQ (feedback de-excitation) and state transitions occur in conjunction with dynamic changes in antenna size in acclimation responses to, *inter alia*, changes in light conditions [19]. More recently, the less abundant LHCI proteins Lhca5 and Lhca6 have been implicated in the regulation of cyclic electron transport [41], which is known to be subject to both evolutionary adaptation and environmental acclimation [see e.g. [42]]. Comparative studies of plants from diverse taxa, ecological niches or habitats (field or laboratory) show that the regulatory properties of the antenna systems typically vary more than the properties of the "core engine" of the system [43].

Twenty-five years ago, *Arabidopsis *emerged as the prime model organism for plant biology research [44]. Its small size and rapid growth cycle has enabled photosynthesis researchers to move from experiments with "synthetic" (e.g. algal cultures) or imprecise (e.g. spinach) systems to more reproducible experiments with plants grown under highly controlled and reproducible conditions in climate chambers, growth rooms and cabinets. Although this has been important for scientific development, we believe that studies performed with plants grown under natural conditions can provide valuable complementary information. This study is a contribution to the growing body of literature describing experiments in which *Arabidopsis *has been exploited as a natural species rather than a "laboratory rat". In the wild, *Arabidopsis *grows in open, typically highly-disturbed, habitats and has significant capacity for photosynthetic acclimation [12]. Therefore, whether or not *Arabidopsis *plants grown in climate chambers are like those grown in the field, which may seem trivial, is highly relevant for scientists addressing many aspects of plant biology. One aspect not covered in this work is the natural variation of the species; it is possible that our results may have been substantially different had we chosen to study a different accession rather than Colombia-0. We chose this accession because it has been used in most studies published to date; however this accession is not specifically adapted to our local study site environment in Umeå. Furthermore, as we have not compared plants grown at different sites, at different times of the year or with different photoperiods we cannot draw general conclusions about the plasticity of *Arabidopsis *in all environments. Additional phenotypic variations may be encountered in future experiments, and we make no claim that other field-grown *Arabidopsis *plants will necessarily be similar to those analyzed here. Nevertheless, we believe that the trends we have recorded are likely to represent some of the most prominent differences between indoor- and field-grown *Arabidopsis *plants. It is also obvious that plants grown in climate chamber under LD are better substitutes for field-grown plants than plants grown under SD--which is typically used for photosynthetic studies--although plants grown indoors under LD were still more similar in terms of photosynthetic characteristics to SD plants than to field-grown specimens (Table 3). Further studies are needed to determine if variations in LD conditions in climate chambers (e.g. 14, 16, 18 or 20 h photoperiods) significantly influence photosynthetic characteristics.

Our data show that the indoor-grown SD plants had, for example, different leaf morphology, higher levels of Lhca5, much higher levels of Lhcb1 and Lhcb2, less PsbS (and no ELIPs), and different pigment contents compared to the field-grown plants. In particular they were strongly depleted in xanthophyll cycle pigments. The differences in leaf morphology and plant stature are striking, and it is intriguing that some of the observed changes, for example in leaf size, did not follow simple patterns, notably both LL- and field-grown plants had smaller leaves than NL- and HL-grown plants. This indicates leaf developmental patterns are influenced by more than one factor. For example, several "typical photoreceptors" may respond both to differences in photoperiod and light intensity, and photosynthetic signals may influence leaf morphology. Accordingly, anatomical differences between typical sun and shade leaves seem to depend on photosynthetic signals [45]. We also found that Lhca5, expression of which correlates well with light intensity in indoor plants, was almost undetectable in the field-grown plants. It appears, therefore, that (at least in *Arabidopsis*) Lhca5 is not simply a "light stress LHC", as exemplified by LI818 in *Chlamydomonas *[46], since it was down-regulated under our field conditions. It has been suggested that both the Lhca6 protein, which is present at very low levels in plants grown under most conditions, and Lhca5 regulate cyclic electron transport around PSI [41]. However, we are not aware of any published analyses of the cyclic electron transport capacity of field-grown plants.

Most or all PSI and PSII core proteins are present in unit stoichiometry and this also probably applies to the PSI antenna proteins Lhca1-4 and the minor Lhcb antenna proteins Lhcb3-6. Our data show that the PSI antenna in the plants grown indoors was similar to that of the plants grown under field conditions but--as we have noted before--the PSII antenna may be more flexible. On a PSII basis, the levels of Lhcb5 (CP26) and, in particular, Lhcb6 (CP24) were lower in indoor plants, raising questions whether PSII centers lacked these proteins in the indoor plants, or a fraction of the proteins was present, but they were not bound in their "normal positions" in PSII in the field-grown plants (or both). Our results relating to the major LHCII proteins (Lhcb1, Lhcb2 and Lhcb3) are particularly intriguing. Taking known pigment and protein stoichiometries into account, there may have been three to four LHCII trimers per PSII monomer in the LL plants. The supermolecular structure of PSII has been studied extensively, and it is known that up to three LHCII trimers, denoted S, M and L, can associate with each PSII complex in a dimer [47]. S, M and L refer to strongly, medium and loosely bound trimers, respectively. It is possible that the M trimer is composed of Lhcb3 and two Lhcb1 subunits [48]. It is not known if there is any specificity for Lhcb1 and Lhcb2 at any position in the S and L trimers. It is conceivable that other LHCII trimers may aggregate in "LHCII-only domains", which must be attached to the photosystems, since energy transfer from all parts of the LHCII antenna into the photosystems is very efficient. Naïvely, the S, M and L trimers plus trimers found in LHCII-only domains may account for three to four trimers/PSII in LL plants. However, the field-grown plants contained only ca. a third of this amount of LHCII, i.e. one or at most two trimers/PSII. Lhcb3 was present in approximately equal amounts in field-grown and indoor plants, suggesting that M trimers were present in most or all of their PSII centers. Our data show that plants with only small amounts of LHCII trimers are perfectly capable of performing state transitions, consistent with the finding that the fitness of the *Stn7 *mutant grown under field conditions deviates from that of wild-type counterparts [7]. However, since the M trimer--at least Lhcb3--is not believed to participate in state transition [47], Lhcb1 and Lhcb2 in S trimers are likely to be efficiently phosphorylated and participate in state transitions in field-grown plants. Alternatively, M trimers may become phosphorylated and detach from PSII. There are insufficient data from our study to enable us to confirm this possibility, but a more detailed study of PSII in *Arabidopsis *grown under field conditions may show which PSII supercomplexes are most abundant when *Arabidopsi*s is exposed to its naturally-adapted light regimes. Taken together, although the LHCII content is much lower in field grown plants, antenna function is not much affected.

ELIPs, most likely involved in pigment metabolism in plastids, were originally identified as proteins that transiently accumulate during early plastid development, but subsequent studies have shown that they also accumulate under diverse stress conditions [29]. ELIPs play an important protective role under light-saturated conditions, such as may occur in the field and, except in some artificially-controlled growth conditions in climate chambers; they are likely to be abundant thylakoid proteins. Nevertheless, our results indicate that the plants lacking ELIPs were well adapted to their growth conditions and had high levels of fitness; our 2-year study of double ELIP mutants suggests that ELIP functions in mature leaves may be redundant or of low importance. However, ELIPs may be more important in early developmental stages and it is also possible that they play crucial roles under conditions that the plants did not encounter during these 2 years.

Xanthophyll cycle pigments and PsbS are typically involved in photoprotective processes. In our experiments these factors were found at very low levels in indoor plants compared with field-grown samples. This is consistent with the view that under natural conditions photoprotection by NPQ and other mechanisms is of vital importance for the fitness of the plant [8]. We have also shown that the level of NPQ is balanced and there is some evidence that selective forces act to reduce the level of photoprotection [9]. Finally, our comparison of NPQ levels in a set of *Arabidopsis *accessions grown in the lab and the field illustrates how conclusions drawn from studies in the lab may be invalid for field-grown plants, due to phenotypic plasticity.

Plants have evolved many mechanisms that are involved in responses to changes in their growth conditions, ranging from long-term developmental processes that affect the morphology or physiology of the whole plant or individual leaves [25,49], to adjustments in the functioning of individual proteins within the photosynthetic apparatus, operating on timescales ranging from seconds to hours [50]. We have studied some of these adjustments, in particular relating to the functions of the photosynthetic light harvesting apparatus. In addition, adjustments to PSI/PSII ratios, variations in components of the inter-photosystem energy flow apparatus, and rates of cyclic electron transport, ATP generation and the photosynthetic dark reactions may be as important as those investigated here. We anticipate that other studies will focus on comparisons of photosynthetic properties that vary between and within species, or in single genotypes, as a result of phenotypic plasticity.

## Methods

### Plant material and growth conditions

Wild type *Arabidopsis thaliana *(Col-0) plants were grown from seeds under short photoperiods indoors under three growth irradiances: 30 (LL) and 300 (NL) μmol quanta m^-2 ^s^-1 ^in growth chambers equipped with metal halide lamps maintained at 8 h light, 16 h dark, 23/18°C and 75% relative humidity; and 600 (HL) μmol quanta m^-2 ^s^-1 ^in a chamber maintained at 9 h light, 15 h dark, 23/18°C and 75% relative humidity. LD indoor conditions were the same as HL conditions, except that the photoperiod was 16 h light, 8 h dark. In addition, another set were prepared and grown in the field as described by [7], as follows. After stratification, seeds were sown on June 29 2009, and seedlings were transferred to individual pots 10 days later on July 9 and pre-grown as above in a NL growth chamber. The resulting plants were transferred to our experimental garden in Umeå (N 63° 49' 9.96" E 20° 18') on July 22, when they had three to four leaves. The plants were shaded on the first day to allow for some acclimation. Photon flux density (PPFD) was monitored at the field site and ranged from very low levels up to 600 W/m^2 ^(ca 2 300 μmol quanta m^--2 ^s^--1^) during the photoperiods, which in the beginning of the experiment was ca. 20 h. The mid-day temperature varied between 16° and 28°C, and the relative humidity (RH) between 30 and 100%. A detailed description of the growing conditions is presented in Additional file 1: Figure S2 (A and B). Fluorescence data were recorded on individual plants in randomized order on August 8; measurements started around 10 am and finished around 6 pm. On August 10 at approximately noon all leaves from the plants used for fluorescence measurements were sampled for pigment and thylakoid protein analysis. Three sample pools, each consisting of leaves from 5 to 15 plants, were sampled and analyzed. The measurement and sampling schemes for LL, NL and HL plants were similar to those applied to plants grown under field conditions. Timings were adjusted to the growth rates under the different conditions, since the intention was to sample plants at similar developmental stages (before bolting), rather than those of the same age. Since the plants were small when transferred to the field but grew considerably before sampling, most of the leaf biomass analyzed consisted of leaves that had developed under field conditions.

We had performed a pilot experiment in the same garden in the summer (2008) prior to the study described above, in which we analyzed the plants' pigment and protein levels less comprehensively. The trends obtained were largely comparable to those found in the main study (data not shown). The plants used for measuring state transitions were grown in the summer of 2010. The chl levels of these plants were monitored to confirm that the size of the light-harvesting antenna was similar to that of the plants grown in 2009.

For the study of NPQ variation, we obtained 14 *Arabidopsis *accessions from the Nottingham Arabidopsis Stock Centre: Van-0, Can-0, Kas-1, Ws-2, Col-0, UK, Sf-2, Old-2, Mt-0, Br-0, Aa-0, Cvi-0, Mr- and Ron-0. We also included two Swedish accessions, and finally a mutant (*npq4*, Li et al., 2000) and a transgenic (*oePsbS*, Li et al., 2002) with varying levels of PsbS and, hence, NPQ. These lines were grown under two different conditions. First, plants were grown in a climate chamber (under NL) conditions as described above and NPQ was measured after 4 weeks of growth. A second batch of plants were grown under the same conditions for 6 weeks, then transferred to the field and measured 5 days later. In both experiments, the different genotypes were grown in a randomized pattern, to avoid misinterpretations of data due to local variations in (for example) light conditions; six plants of each genotype were analyzed.

### Leaf size and shape

Leaf shape and size were quantified using the imaging software LAMINA [51].

### Chlorophyll determination

Chlorophylls were extracted from leaf tissue with 80% (*v/v*) acetone and assayed spectrophotometrically using extinction coefficients according to [52].

### Carotenoid analysis

Carotenoid composition was determined by high-pressure liquid chromatography (HPLC) [53] with modifications described by [49]. The de-epoxidation state of the xanthophyll pool was calculated as (Z + A/2)/(V+A+Z) where V = [Violaxanthin], A = [Antheraxanthin] and Z = [Zeaxanthin].

### Chlorophyll fluorescence and state transition measurements

Chlorophyll fluorescence of the plants was measured, after dark-adaptation, with a Dual PAM 100 chlorophyll fluorescence photosynthesis analyzer (Heinz Walz) as previously described [48]. For NPQ measurements, actinic illumination was 660 μmol photons m^-2 ^s^-1 ^for 20 min, followed by darkness. A saturating pulse of light (5000 μmol photons m^-2 ^s^-1^) was given every 1-2 min.

### Immunoblot analysis of thylakoid membrane proteins

Immunoblot analysis of thylakoid membrane proteins was performed as described by [54], with modifications. Five- to six-week-old leaves were homogenized and filtered using a nylon mesh with a 20 μm mesh size (Millipore). The filtered homogenate was pelleted and resuspended in hypotonic buffer to break the chloroplasts. The thylakoid membranes were pelleted then resuspended in 0.33 M sorbitol, 20 mM Tricin (pH 7.8) and 5 mM MgCl_2_. All of the preparation steps were performed on ice or in a cold room (4^°^C) under a green safe light. Thylakoid proteins were prepared for immunoblot analysis by addition of Laemmli denaturation buffer [55] and incubation at 90^°^C for 10 min [53]. One microgram of chlorophyll was loaded per lane, and the proteins were separated in a 16% denaturing SDS-PAGE gel (with non-urea buffers) using the Bio-Rad Mini Protean III system. The proteins were blotted on nitrocellulose membranes (Bio-Rad; 0.2 μm), using a Bio-Rad wet blotting system with methanol-containing buffers, according to the manufacturer's instructions. The nitrocellulose membranes were blocked using 5% (*w/v*) non-fat dried milk in TBS-T buffer with 0.1% Tween 20 for 1 h (Sigma-Aldrich Sweden AB) and incubated using rabbit primary antibodies against photosynthetic proteins [54,56,57] (provided by Agrisera, Vännäs, Sweden) at 1:5000 dilution for all antibodies (except anti-Lhca5 antibody, which was diluted 1:2000), for 1 h in TBS-T buffer with 0.1% Tween 20 and 5% non-fat dried milk. The membranes were washed three times for 5 min in TBS-T buffer, 0.05% Tween 20 and incubated with anti-rabbit donkey antibody horseradish peroxidase (HRP) conjugate (GE Healthcare Bio- Sciences) for 1 h at 1:10,000 dilution in TBS-T buffer with 0.1% Tween 20 and 5% non-fat dried milk. Immunoblotted membranes were incubated for 2 min in ECL plus HRP substrate (GE Healthcare Bio-Sciences), and chemoluminescence was then detected using a LAS-3000 cooled CCD camera. Optimal exposure times ranged from 5 to 10 min, and identical exposure times were used to quantify signals for each antibody used. Images were recorded using Image Reader software with 1 min incremental recording and standard CCD sensitivity (Fujifilm Medical Systems). The images were processed and quantified by the Multi Gauge application (Fujifilm Medical Systems), using profile lane quantification with automatic background subtraction and band detection. Standard parameters for peak detection were used according to the manufacturer's instructions.

### Statistical analysis

Results were statistically analyzed using one-way ANOVA implemented in SPSS18 software applying Duncan's new multiple range tests to analyze all possible differences between LL, NL, HL and field plants. In addition, an orthogonal contrast analysis was done to see the difference between indoor and field plants (contrast indoor vs. field plants). The number of independent variables for each experiment was three.

## Authors' contributions

YM, CF, WS, and SJ conceived and designed the experiments. YM, AZK and HJJ performed the experiments. YM, AZK and HJJ analyzed the data. YM, WS and SJ wrote the paper. All authors discussed results and commented on the manuscript.

## Supplementary Material

Additional file 1 **Figure S1 State transition in LL, NL, HL and field grown plants**. Average room temperature fluorescence traces. The black bar below the trace indicates far-red light OFF (state 2 inducing) treatment and the gray bar below the trace indicates far red light ON treatment (state 1 inducing). **Figure S2 Weather conditions in Umeå when the plants were grown during the field experiments. (**A) August 2009 and (B) July 2010. Source: http://www8.tfe.umu.se. Click here for file
