# Supplementary figures and images for: Arabidopsis plants grown in the field and climate chambers significantly differ in leaf morphology and photosystem components
Source: BMC Plant Biol. 2012 Jan 11;12:6. doi: 10.1186/1471-2229-12-6 (PMC3296669; doi:10.1186/1471-2229-12-6)

The weather in August 2009

Additional file 1. (A)

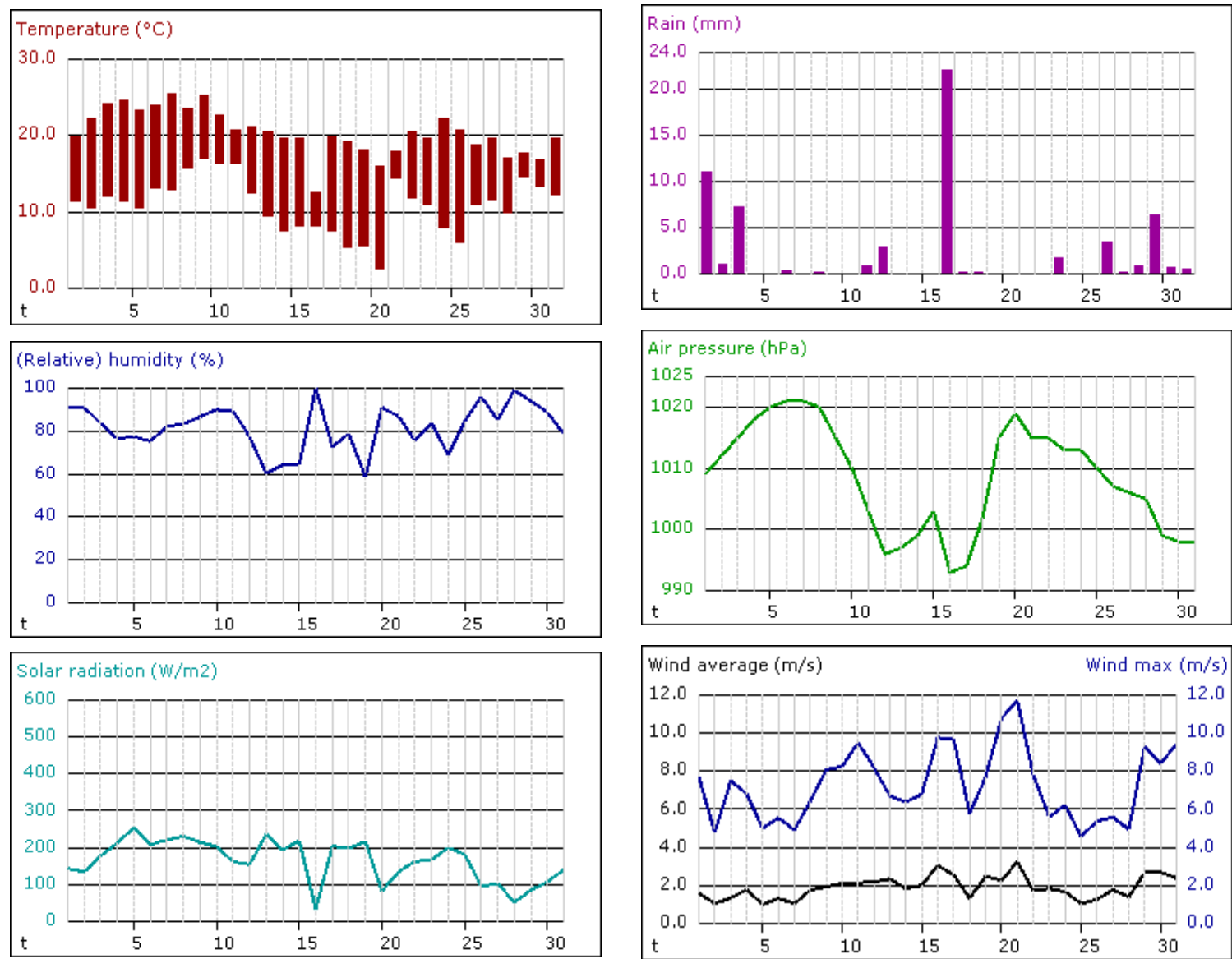

The weather in July 2010

(B)

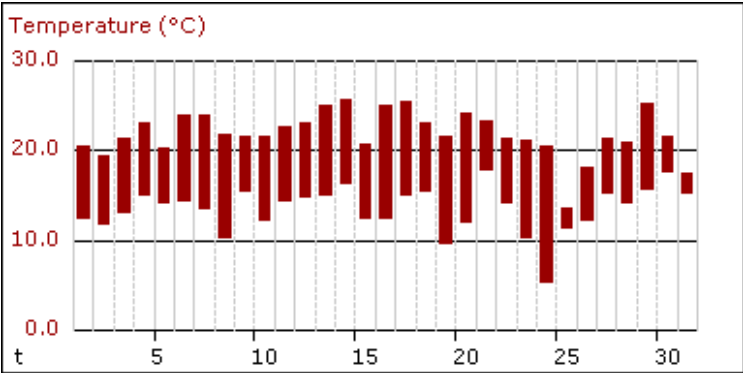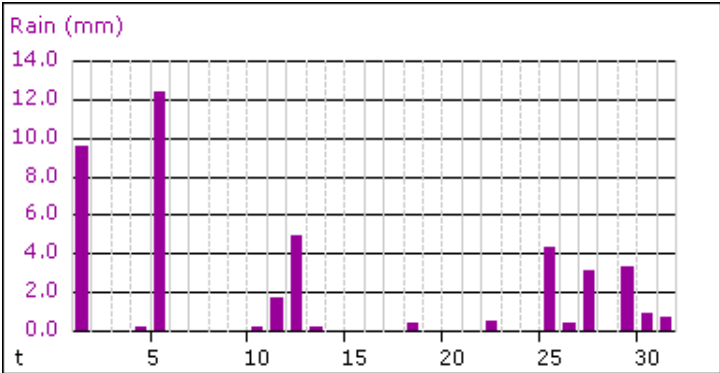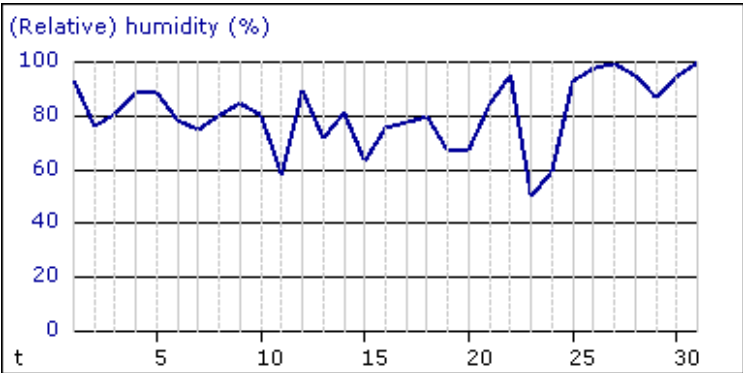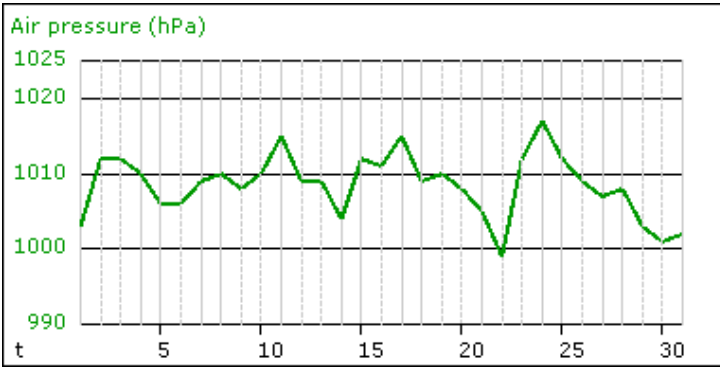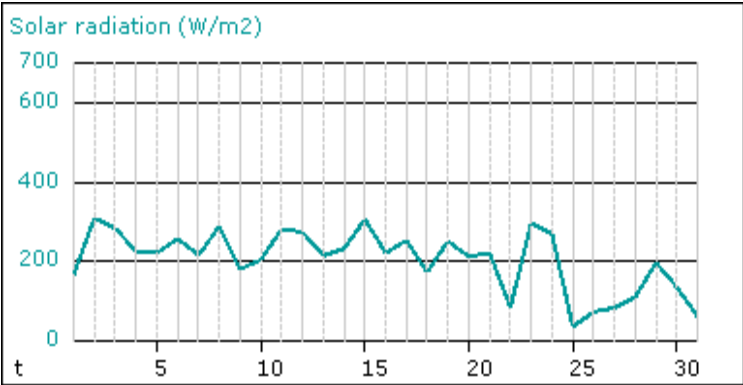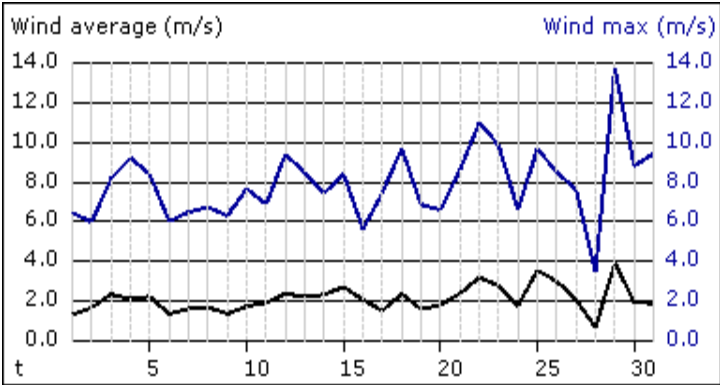

Additional file 2.

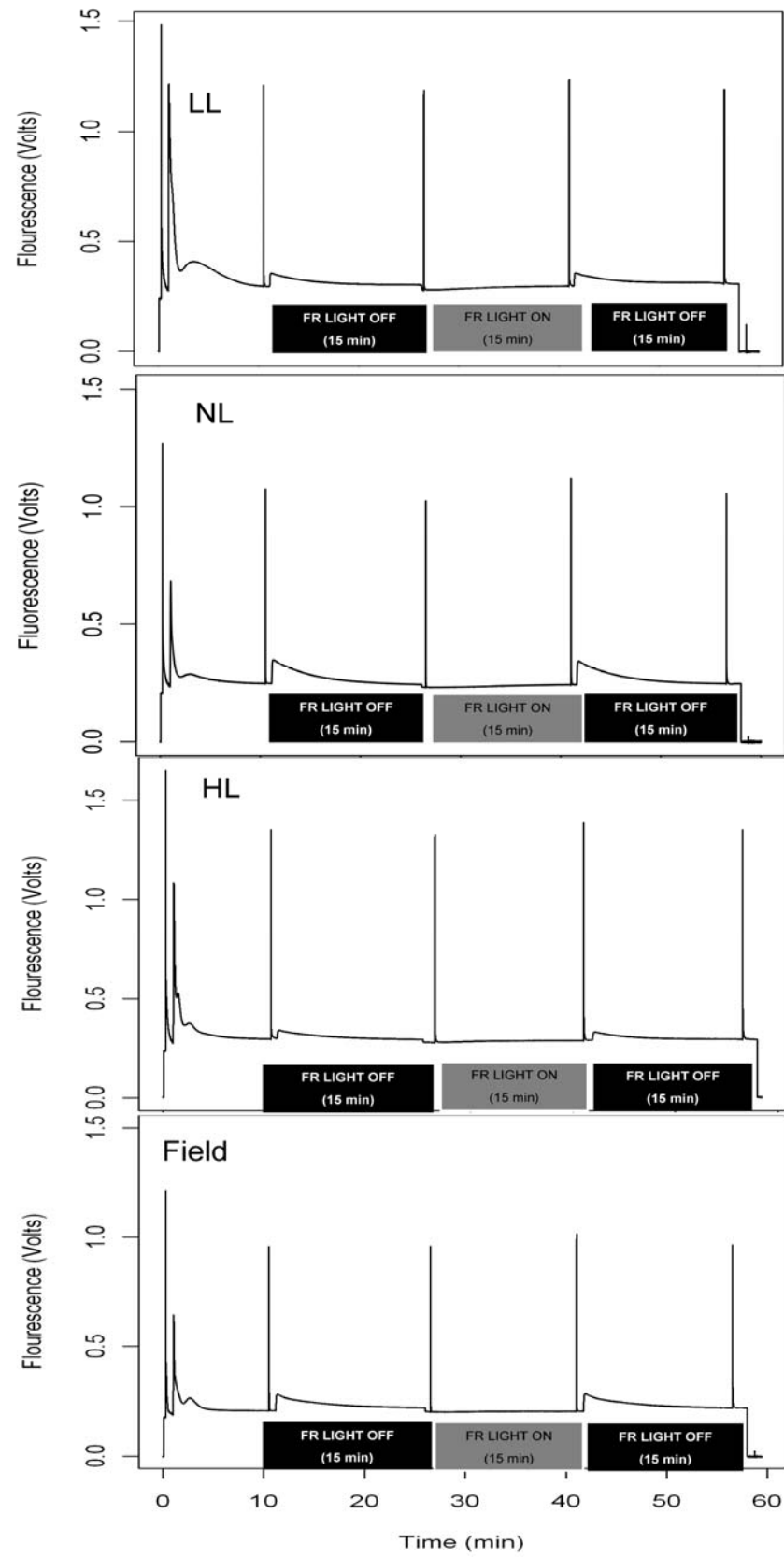

Supplement: Additional file 1 — Figure S1 State transition in LL, NL, HL and field grown plants. Average room temperature fluorescence traces. The black bar below the trace indicates far-red light OFF (state 2 inducing) treatment and the gray bar below the trace indicates far red light ON treatment (state 1 inducing). Figure S2 Weather conditions in Umeå when the plants were grown during the field experiments. (A) August 2009 and (B) July 2010. Source: http://www8.tfe.umu.se. [file 1471-2229-12-6-S1.PDF]
